# Allegheny County Medical Society

**Published:** 1889-10

**Authors:** James McCann

**Affiliations:** In the Chair


### Society Reports.

### ALLEGHENY COUNTY MEDICAL SOCIETY.

*Special Meeting August 20th, 1889.*

JAMES McCANN, M. D., IN THE CHAIR.

### TREATMENT OF PULMONARY PHTHISIS.

Dr. Lange reported a new method for the treatment of pulmonary phthisis. The method consists in the inhalation of vaporized mercury and iodine. Of the results of this method I have nothing to say. I cannot forget that grass has not yet grown upon the grave of gaseous enemata, and I am aware that many men, many years and many cases are required to produce evidence of the usefulness of any remedy or method in the treatment of anything, even when the remedy or method possesses usefulness. I report this method because I desire co-workers.

It suggested itself to me that vaporized mercury, if brought into more or less direct contact with the bacillus of Koch, might destroy this, and that iodine, if applied directly to the ulcerating surfaces of lung tissues, might effect a more powerful beneficial action than that resulting from its ordinary method of administration. I have had, and still have, the valuable assistance of Dr. Tingley in the preparation of apparatus and in devising ways and means by which these vapors may be satisfactorily administered to patients. This has presented many difficulties. A principal one is that I know of no manner, as yet, by which a difinite, a known quantity can be given. The vaporized mercury salts are resublimed and deposited upon the cooler parts of the apparatus. This is particularly true of the inhaling tube, which is always the coolest part of the apparatus. The consequence of this is that patients receive an unmeasured, an accidental quantity of these salts or vapors, and not a quantity which is measured or known. To this fact are due two accidents, namely, that one very feeble patient was violently purged and another was salivated. However, we hope to overcome this defect of apparatus and to be able soon to give patients exact quantities of these salts. The desideratum is an inhaling tube which will bear the temperature necessary to hold the mercury salts vaporized up to the lips of the patient, and which at the same time shall be flexible. Fexibility is almost a necessity; a feeble patient cannot breathe deeply and persistently from a stiff tube, a glass tube, such as I now use.

I have found that the only salts of mercury available for this purpose are the red oxide and calomel. All others are reduced before being volatilized. I began with the iodide of mercury. This and all others, when used, result in the vapor of metallic mercury only. I have found no objection, however, to the use of metallic mercury, only it is to be noted that when other salts than calomel and the red oxide are used, the patient receives the vapor of metallic mercury.

Can the vapor of mercury, or can anything inhaled, reach the bacilli in a tuberculous lung? Those bacilli, which are in consolidations, provided such a consolidation is connected with a previous bronchial tube, those in lung cavities furnished in the same manner, those in the bronchial tubes, those in the alveoli, and those in the sputum may be reached by this vapor or by anything which may be deeply and persistently inhaled. But these bacilli are comparatively inert; they are harmless; they have already accomplished their mission of destruction and are being extruded from the body. Those whose destruction is very much more desirable; those which have not yet but which certainly will produce consolidation and softening, *i. e.*, destruction of lung tissue; those in the pulmonary connective tissue, and the lymphatic sheaths of the blood vessels, can these be reached by anything that may be inhaled? Again, if we grant that in a certain patient every bacillus has been destroyed, this is by no means synonymous with his cure. Evidences of this fact are presented daily; patients die of non-tuberculous phthisis very readily. And the tuberculous patient with every bacillus in his lungs destroyed, possesses still that fatal predisposition and will be reinfected.

It is a question also whether mercurial vapor is a germicide. No one will deny this property to corrosive sublimate. But corrosive sublimate is not volatilizable, and volatilized mercury, volatilized calomel and red oxide are very different substances indeed.

Despite these theoretical objections I am encouraged to proceed with this treatment of phthisis, and when I have perfected the apparatus and have a series of cases certainly turbercular, as demonstrated by the discovery in the sputum of the bacilli, which Dr. Matson has kindly consented to do for me, I shall report again to the society.

Dr. R. W. Stewart exhibited his

IMPROVED URETHREGRAPA, and announced that it was now as nearly perfect as he could make it, and that he had made arrangements by which the profession could be supplied. As at present constructed the instrument can be introduced within the bladder,and by merely withdrawing it, it will give a tracing of the entire length of the urethra. He exhibited tracings taken from a single patient under the care of Dr. Carrington, the first before operation and the second after operation with Holt's divulsor. The result of the operation was evident on the tracings.

Dr. J. D. Thomas complimented Dr. Stewart on the construction of the instrument, and stated that he had long since recognized the defects in Dr. Otis' urethrometer; it was too stiff; the distal side as well as the distal portion of the stricture was easily enough detected and measured, but as the "meter" was brought forward the variations in the calibre and the proximal termination of the stricture required very delicate manipulation and much experience, to gain the desired information. In this connection Dr. Thomas exhibited to the Society an improved urethrotome of his own invention. In his experience the Otis urethrotome had three defects: 1st. After the blade was drawn forward and the stricture divided it was necessary to push the blade back into its sheath or recess and thus, although not intentional, make two incisions—the second one likely to be a lacerated one. 2d. If more than one stricture existed the instrument had to be adjusted for each stricture, thus causing unnecessary irritation of the urethra; and 3d, as the instrument opened on the principle of a parallel ruler, there was more or less displacement after the instrument was opened. Dr. Thomas demonstrated the working of his urethrotome, and showed that by its use the above objections were overcome. The instrument of Dr. Thomas is made by Tiemann & Co., of New York.

Dr. Werder read a paper reporting a case of

FIBRO-CYSTIC TUMOR OF THE UTERUS.

(See page 463.)

Dr. J. J. Buchanan: This operation at which I was present, was a very difficult and interesting one. From the appearance of the patient prior to operation, I do not believe that it would have been possible for any one to have made a diagnosis other than ovarian cystoma. The fluctuation was as distinct as though the tumor had been a single sac filled with fluid. The tumor was markedly at one side of the abdomen. It lay in front of the uterus, and in every respect that I can now think of, it exactly imitated the behavior of the ordinary ovarian cyst. With regard to the pathology, I think it rather unfortunate that there was no microscopical examination made. As to the treatment, I think it was everything that could be desired, as the result proved, although I believe that a large proportion of these cases when so treated are fatal on account of the extensive amount of tissue which has to be left to slough in the neighborhood of the peritoneal cavity. The suggestion of Dr. Werder that probably the enlargement of this growth midway between the menstrual periods was coincident with the period of ovulation, I think is hardly susceptible of proof.

Dr. Blume: Doctor Werder is to be congratulated for his successful operation. His case offers some interesting points as regards diagnosis and treatment. All authorities tell us that an exact diagnosis in cases of large abdominal tumors often is impossible, and every one who has had an opportunity to examine such cases will confirm this. I desire to call your attention to a diagnostic symptom which has not yet been mentioned in our text books. Recent investigations by competent men have proven, that in all cases of uterine tumors the endometrium is diseased. Clinical experience has taught this long ago. Hemorrhage in form of menorrhagia or metrorrhagia is known to be often a prominent symptom of fibroma and myoma. The endometritis in these cases is usually the consequence of the growth and therefore secondary. In diseases of the ovaries—oophoritis and cystic ovaries, as well as in tubal diseases--endometritis is constantly met with, and here often as a primary malady. Malignant growths of the ovaries-sarcoma and carcinoma are also complicated with a diseased endometrium, while this complication is seldom found in cases of ovarian cystoma.

Although there are patients with large fibroma or myoma, who never lose an excessive amount of blood, and the case under discussion is an example, endometritis nevertheless always exists as a complication.

I therefore suggest, that in all cases of large abdominal tumors, after all diagnostic means to arrive at an exact diagnosis are tried in vain, the endometrium should carefully be examined. If endometritis is found to be present, the tumor may originate from the uterus. If the endometrium is found to be healthy, the tumor is proven not to be in connection with the uterus.

The treatment resorted to in this case appears to be somewhat different from the usual *modus operandi*. The tumor, if I understood the doctor right, was a myoma interstitialis, developing from the fundus and right uterine wall. The ligaments were tied, the tumor cut away and a large pedicle treated extraperitoneally. Tumors of this kind are either enucleated and the uterine walls stitched together, or removed by supravaginal amputation, the pedicle treated either extra or extraperitoneally. The extraperitoneal method, strongly advocated by Hegar and Kaltenbach, has given the best results. Kaltenbach lost one of twenty-two cases, equal to 4½ per cent; Keth, two of thirty-eight, equal to 5¾ per cent. I am in doubt, like Doctor Buchanan, whether or not it is indifferent to form a thick pedicle, as was done in this case.

A few months ago I saw a similar operation performed by Dr. Burns. The tumor, a large fibroma, broadly attached to the fundus uteri, was not enucleated but removed by a wedge-shaped incision. The remaining portion was carefully sewed and the patient made a rapid recovery.

